# The Impact of an *Intensive English Reading* Course Based on the Production-Oriented Approach on the L2 Motivational Self System Among Chinese University English Majors From a Dynamic Systems Theory Perspective

**DOI:** 10.3389/fpsyg.2021.761093

**Published:** 2022-01-03

**Authors:** Chili Li, Chujia Zhou, Wen Zhang

**Affiliations:** ^1^School of Foreign Languages, Hubei University of Technology, Wuhan, China; ^2^New Channel Wuhan School, Wuhan, China; ^3^Jianghan Oilfield Senior Middle School, Qianjiang, China

**Keywords:** *Intensive English Reading* course, Chinese university English majors, Production-Oriented Approach, L2 Motivational Self System, Dynamic Systems Theory

## Abstract

This article reports on a study that took a Dynamic Systems Theory (DST) perspective to second language (L2) motivational self system (L2MSS). More specifically, it investigated the influence of an *Intensive English Reading* course based on the Production-Oriented Approach (POA) upon the L2MSS of Chinese university English majorsfrom the DST perspective. To this end, two intact classes composed of 50 students were assigned into experimental group (EG) (*N* = 23) and control group (CG) (*N* = 27), who responded to an L2MSS scale before and after the one-semester intervention. Eight and five students were respectively selected using the purposive sampling method from the experimental and control groups for follow-up semi-structured interviews. The quantitative results revealed that the overall and dimensional (Ideal L2 Self and L2 Learning Experience) levels of L2MSS were significantly strengthened over time in the EG while kept stable in the CG. The qualitative results suggested that the enhanced Ideal L2 Self of the participants stemmed from an attractor basin that was deepened by a number of attractors encompassing *Output Tasks* and *Peer Performance*. The interview results also showed that the increased L2 Learning Experience of the participants pertained to an attractor basin that was consolidated by an array of attractors containing *Output Tasks*, *Teacher Guidance*, *Group Discussion*, and *Peer Assessment*. The findings indicated that the attractors at the subjective and social dimensions in the POA-based course collectively worked together to cause changes in L2MSS among the participants. The implications for intervening L2 motivation from a POA approach in English as a Foreign Language (EFL) classrooms were discussed.

## Introduction

Second language motivation is one of the critical learner variables that considerably influence the success of second language (L2) learning (Dörnyei, 2014). Since Gardner and his associates (i.e., Gardner and Lambert, 1959) pioneered the instrumental-integrative dichotomy, L2 motivational research has undergone a number of stages, namely, social-psychological, cognitive-situational, and process-based orientations (Dörnyei and Ushioda, 2009). The early perspectives mainly follow the linear paradigm to examine the collective features of L2 motivation (Chang, 2019) and explore the relationship between L2 motivation and other learner variables (Zhan, 2015). However, the linear paradigm fails to situate the learners into specific contexts (Dörnyei et al., 2015). This paradigm also pays little attention to the complexity of the interaction among motivational factors (Xu and Fang, 2016).

In light of the paradigmatic and theoretical shortcomings in the early approaches, a dynamic and non-linear approach has been increasingly called for since the beginning of the new millennium (Dörnyei et al., 2015; Wang et al., 2017). Corresponding to this call are some theoretical efforts, such as the second language (L2) motivational self system (L2MSS) (Dörnyei, 2005). L2MSS advocates a non-linear approach to examining L2 motivation (Dörnyei et al., 2015). This assonates with what is claimed by Dynamic Systems Theory (DST). DST recommends exploring the interactive relationship between systemic development and situational context from a non-linear lens (Larsen-Freeman, 2015; Hiver and Papi, 2019). This suggestion echoes the person-in-context position that L2 motivation should be understood in tandem with specific situations (Dörnyei and Ushioda, 2011). However, the present effort to explore L2 motivation from the DST perspective is mainly at a theoretical level and inadequate in empirical studies (Waninge et al., 2014).

Meanwhile, L2 motivational intervention has been attracting attention from researchers (e.g., Fang and Chen, 2013; Duan, 2020). Studies have shown that L2MSS could be significantly enhanced by pedagogical practice (Wang and Dai, 2015). Teaching modes, such as the POA-based model, could nurture Positive Psychology in L2 learning among learners (Qiu, 2020). These studies indicate a possible connection between Positive Psychology and L2 motivational development (MacIntyre et al., 2015). However, the process for POA to achieve such an effect remains unknown (Chen and Wen, 2020).

The above-mentioned issues, therefore, warrant further research on the impact of teaching modes, such as the POA teaching model, on L2 development. It is of paramount importance to examine how L2MSS changes and what contributes to its dynamics under the POA teaching model. Therefore, this study aims to explore the influence of a POA-based *Intensive English Reading* course on the L2MSS among Chinese university English majors from the DST perspective.

## Literature Review

### Second Language Motivational Self System

Research on L2 motivation has mainly followed a sociodynamic approach over the past two decades (Dörnyei and Ushioda, 2011). This perspective examines L2 motivation from a number of theories, such as L2 Motivational Self System (L2MSS) (Dörnyei, 2005, 2009). L2MSS highlights the necessity of understanding L2 motivation from the perspective of self against specific situational context (Chang, 2018). It consists of three components: Ideal L2 Self, Ought-to L2 Self, and L2 Learning Experience. Ideal L2 Self refers to the external manifestation of ideal L2 learner traits that an individual would like to have, which stimulates the learners to bridge the gap between their ideal self and their actual self. Ought-to L2 Self is associated with qualities that L2 learners believe that they should possess in order to meet external expectations and avoid possible negative consequences. L2 Learning Experience is related to motivation pertinent to the immediate learning environment and experiences, such as teacher influence, curriculum, peer groups, and successes (Dörnyei, 2009). L2MSS takes motivation as a complex dynamic system (Dörnyei et al., 2015).

Previous research has mainly validated L2MSS in various English as a Foreign Language (EFL) contexts, such as China, Iran, and Japan (Taguchi et al., 2009). Ideal L2 Self and L2 Learning Experience mediate the effect of self-efficacy on English proficiency (Huo and Rui, 2020). L2MSS is affected by factors, such as teaching model, and teacher behaviors (Duan, 2020). For instance, a strong correlation is found between social goals and Ideal L2 Self and Ought-to L2 Self of learners (Prasangani, 2015). In addition, interest proves to be a significant variable that contributes to Ideal L2 Self of learners, whereas Ought-to L2 Self tends to be associated with internalized instrumental motivation (Islam et al., 2013). These studies crystallize the structural features as well as influencing factors of L2MSS. However, they mainly adopt normative techniques to explore the L2MSS features and linear relationships between L2MSS and other learner variables (Zhan, 2015). This paradigm is unable to show the non-linear relationship between L2MSS and specific contextual conditions (Dörnyei et al., 2015).

### L2 Motivation From the Dynamic Systems Theory Perspective

Given the paradigmatic and theoretical issues in the early approaches to L2 motivation, the sociodynamic turn from a DST perspective has been called for over the past two decades (Dörnyei et al., 2015; Wang et al., 2017). DST advocates a holistic view to examine how the interaction among the components of a system leads to the interaction between the system and context (Larsen-Freeman and Cameron, 2013; Hiver and Al-Hoorie, 2020).

A dynamic system includes attractors, attractor state, and attractor basin (Larsen-Freeman and Cameron, 2008). Attractor is an important sign of the dynamic change of the system (Larsen-Freeman and Cameron, 2013). Attractor state relates to a relatively stable state in which the system is temporarily stable and develops back and forth (Hiver, 2015). Attractor basin is a set of initial conditions that allow a complex and dynamic system to evolve to a given attractor state. The range of an attractor state decides whether a system could move into an attractor basin (Hiver, 2015). Attractor will cause the system to leave the attractor basin, move to the next attractor, and follow the new attractor into another attractor basin (Larsen-Freeman and Cameron, 2013).

A dynamic system is featured with initial conditions, non-linearity, and self-organization (Waninge et al., 2014). System is sensitive to initial conditions. A subtle change in initial conditions would lead to change in the whole system. This change is not a mere aggregation of changes in each composing element, and is non-linear. Self-organization is simultaneous emergence that makes a system adaptive to the outside milieu (Sampson, 2016). The development of a system stems not only from the interaction of the external surroundings, but from the self-organization of the internal elements of the system (Larsen-Freeman, 2015).

Similarly, the development of L2MSS results from the interaction between individual and social-cultural factors (Dörnyei et al., 2015; Chang, 2018). L2 motivational system is composed of subsystems, such as personal goals, instructional settings, and immediate environment (Csizér and Kormos, 2010). These subsystems are co-adaptively interrelated, with change in one possibly leading to change in another and, altogether, contributing to changes in the whole L2 motivational system. For instance, the interaction among expectations, campus atmosphere, and classroom instruction has been found to be responsible for motivational fluctuations among Chinese EFL learners (Chang, 2017).

In summary, DST advocates a non-linear lens to approach the interaction between system development and contextual situations (Larsen-Freeman, 2015). This stance agrees with the person-in-context position of L2 motivation, which calls for a non-linear perspective to L2MSS (Dörnyei et al., 2015). However, existing research on L2 motivation following the DST approach is mainly at the theoretical level. Thus, more empirical evidence in various contexts is needed (Dörnyei, 2014).

### Dynamic Systems Theory and Positive Psychology

Positive Psychology holds that learner emotion is associated with interactions between internal and external factors (Dewaele et al., 2019). Institutional factors (e.g., school, classroom, and teaching quality) have a collective influence on the affective growth of students (Gabryś-Barker, 2016). Specifically, a classroom environment with harmonious relationships, enjoyment, and mastery goal orientation positively predicts learner commitment and achievement (Frenzel et al., 2018). A positive classroom climate characterized by teacher support and student cohesiveness also yields learner enjoyment (Khajavy et al., 2018). Enjoyment then nurtures a positive orientation and willingness to communicate in class (Teimouri et al., 2020).

These positive psychological features have been found to contribute to L2 motivation. Factors, such as interest (Islam et al., 2013), self-efficacy (Huo and Rui, 2020), and strengthened mastery goal orientation from pedagogical intervention (Prasangani, 2015; Duan, 2020), have been found to be closely related to L2MSS. These features fall into the traits of Positive Psychology, such as resilience and enjoyment (Chen et al., 2020; Wang et al., 2021). These psychological traits are dynamic systems that are responsive to social dimensions through interaction with social agents (De Ruiter et al., 2019) and private dimensions of personal experience of joy, fun, and sense of progress (Elahi Shirvan et al., 2020). This suggests the possibility of examining L2MSS by integrating DST and Positive Psychology (MacIntyre et al., 2015). DST argues that L2 development results from complex, dynamic, self-organizing, and adaptive systems (Larsen-Freeman and Cameron, 2008). To understand L2 development thus requires a holistic approach to considering both contextual conditions and the emotion states of learners (MacIntyre and Mercer, 2014).

### The Production-Oriented-Approach- Based Teaching Model and L2 Motivation

The effectiveness of multiple motivational strategies to intervene learner motivation in L2 class has been recently drawing attention from the academia (e.g., Fang and Chen, 2013). For instance, the Production-Oriented Approach (POA) teaching model has been reported to be effective in strengthening Ideal L2 Self (Widdowson and Seidlhofer, 2018; Duan, 2020). The POA teaching model follows the learning-centered principle and emphasizes the integration of language learning into language application (Wen, 2018). It holds that output-driven tasks could promote language input and foster competence in applying language into meaningful communication. This teaching approach includes a circular chain of teaching procedures from motivating to enabling, and then to assessing stages (Wen, 2018).

Empirical evidence has been documented that the POA teaching model can contribute to learning efficiency (Sun, 2020), L2 development (Chen, 2020), and positive psychological experience, such as interest and motivation (Zhang, 2020). The enjoyment and satisfaction generated from executing and orienting toward the tasks throughout the POA teaching process would form a positive effect, which corresponds to the actualization of well-being in Positive Psychology (Chen et al., 2020). The sense of satisfaction would, in turn, help learners resist demotivation, and regain and maintain motivation (Li, 2021).

In a nutshell, POA has a positive effect on the psychology of learning (Chen and Wen, 2020). It is effective in improving the learning efficiency of learners and enhancing their interest in learning English (Sun, 2020). However, the mechanism of how it does so is unknown (Zhang, 2020). Particularly, how the POA model would affect the L2MSS of learners remains to be examined (Duan, 2020). In light of the issues identified above, this study proposes to explore the effect of the POA teaching model on the L2 motivation of learners from the perspective of DST. Specifically, the study intends to investigate whether and how the POA model can change in L2 Motivational Self System among a cohort of Chinese university English majors in an *Intensive English Reading* course and what attractors might have contributed to this change.

## Research Design

### Research Questions

This study aimed to explore the influence of a POA-based teaching model on L2MSS among a group of English majors (experimental group, EG) in a POA-based *Intensive English Reading* course in contrast to another group of English majors (control group, CG) registered in the same course but taught with the traditional teaching model. It mainly addresses the following questions:

(1) Are there any changes in L2MSS of the EG registered in a POA-based *Intensive English Reading* course? If yes, then how?
(2) What are the attractors in the POA-based *Intensive English Reading* course that might cause the changes in L2MSS among the participants in the EG?

### The *Intensive English Reading* Course From the Production-Oriented Approach Perspective

The POA model was implemented in the EG for one semester. In the motivating session, the teacher assigned the output tasks with communicative scenarios in which the theme of the unit was introduced. These tasks were aimed to combine language learning and language application. Students were supposed to complete the output tasks that were designed to moderately go beyond the current level of knowledge and language skills of the students. The challenge to complete the task would, thus, create a sense of “hunger” that would motivate the students to learn. In the enabling session, the teacher divided one output task into several subtasks and provided supplementary materials for the students to learn selectively in order to complete the task. In the assessing session, the teacher demonstrated the objectives and requirements, and demonstrated assessment steps to the students. Then, the teacher asked them to evaluate the production work of each group. They were supposed to think independently first and then discuss in groups. Finally, the teacher encouraged each group to report their evaluation in class. After class, the students were required to revise their work based on the comments they received from other peers in the peer assessment session in class, and then they were asked to resubmit their work to the teacher.

In contrast, the traditional teaching model was implemented in the CG. Before reading, the teacher usually introduced first the background knowledge of the text to the students. During reading, the teacher provided comprehension questions to stimulate the thinking of the students. Then, the teacher checked the answers of the students to these questions. This was followed by the explanation of the teacher of important language points of the text. After reading, the students were required to complete an after-school exercise that would help reinforce their mastery of the language points they had learned.

Sharp differences can be observed between the traditional and POA models in teaching ideas and processes. In the traditional teaching model, the teacher dominated the class. Relying on the textbook, teaching was based on the lecture of the teacher. The POA-based class, on the other hand, advocated a learning-centered principle and emphasized the integration of language learning and use. It provided output tasks to the students, so that they could apply what they had learned. In terms of teaching process, the traditional *Intensive English Reading* class followed a procedure of pre-reading, during-reading, and post-reading, while the POA-based class was presented with a process from motivating, to enabling, and then to accessing.

### Participants

#### Participants for the Questionnaire Survey

The EG involved 23 participants, namely, 2 males and 21 females. The average score on their final exam in the *Intensive English Reading* course from the previous semester was 86.96 out of 100 in total. Ten of them were from urban areas, eight from towns, and five from the countryside. As for their English proficiency, no one rated themselves to be at a high level; 18 students considered themselves to be at a medium level; and the remaining 5 assessed themselves to be at a low level.

The CG included 27 participants with 4 males and 23 females. The average score on their final exam in the course from the previous semester was 85.41 out of 100 in total. Twelve of them originated from cities, seven from towns, and eight from rural areas. Like in the EG, no one in the CG considered themselves to be advanced English learners. Twenty-three of them self-assessed themselves to be at an intermediate level, while 4 rated themselves to be at a low level.

It can be seen that the experimental and control groups shared a similar background in terms of gender, hometown, and self-assessment of English proficiency. They also showed no statistical differences on their final exam scores in the *Intensive English Reading* course from the previous semester (*t* = 1.38, *p* = 0.174 > 0.05). That is, the two groups were at the same level of English language proficiency in the beginning of the experiment.

#### Participants for the Semi-Structured Interview

Eight participants from the EG and five participants from the CG, using a purposive sampling method (Dörnyei, 2007), were invited for a semi-structure interview in order to explore the potential attractors that might have caused the changes in their L2 motivation when studying in the POA-based *Intensive English Reading* course. Both groups did not have any exposure to the POA model before.

All the interviewees were pseudonymously named. Seven interviewees of the EG were females (students A, B, C, D, E, F, and G), and one was male (student H). The average score on their final exam in the *Intensive English Reading* course from the previous semester was 87.13 out of 100. Two of them came from the countryside, five from towns, and one from an urban area. Six of them self-evaluated themselves to be at an intermediate level, and the other two considered themselves to be at a low level in terms of English language proficiency.

All five interviewees (students I, J, K, L, and M) of the CG were females. The average score on their final exam in the *Intensive English Reading* course from the previous semester was 80 out of 100. Two of them came from the countryside, two from towns, and one from cities. Regarding their English proficiency, three of them considered themselves at a medium level, and the other two perceived themselves to be at a low level.

### Instruments

#### Questionnaire

A 20-item L2MSS questionnaire, by drawing on sources from You and Dörnyei (2016) was designed to collect the quantitative data for this study. Before it was finalized, six English majors were invited for a pilot study. Based on their feedback, a revision was further made. The questionnaire consists of two sections. Section One aimed to elicit the demographic information of the participants regarding their gender, origin, scores on the final exam in the *Intensive English Reading* course, and self-perceived English language proficiency. Section Two investigated the Ideal L2 Self of the participants (items 2, 4, 7, 10, 13, 15, 16, and 19), Ought-to L2 Self (items 1, 5, 8, 12, 14, 18, and 20), and L2 Learning Experience (items 3, 6, 9, 11, and 17). The questionnaire followed a 5-point Likert scale, ranging from *Strongly Disagree* (1) to *Strongly Agree* (5).

The Cronbach alphas of the L2MSS scale for the EG before and after the experiment are 0.810 and 0.805 respectively, with those for the three dimensions ranging from 0.604 to 0.808. As for the CG, the Cronbach alpha of the L2MSS is 0.749, with corresponding figures for the three dimensions ranging from 0.672 to 0.733 before the experiment. The Cronbach alpha for the whole scale is 0.755, and those for the three subgroups ranged from 0.71 to 0.754 after the experiment. These figures show that the instrument has moderately good internal consistency and reasonable reliability.

#### Semi-Structured Interview

The interview protocol was adapted from previous studies (e.g., Sun, 2020; Zhang, 2020). It was piloted by five English major students before the protocol was finalized. The interview protocol included two sections. The first section inquired the background information of the interviewees, such as their gender, origin, scores on the final exam in the *Intensive English Reading* course, and self-rated English language proficiency.

Section Two aimed to figure out the factors that might have caused changes in L2 motivation among the participants in the POA-based *Intensive English Reading* course. It contained questions regarding the most memorable classroom POA activities of the interviewees, challenges they encountered in fulfilling these activities, most impressive classmates, feedback, and evaluation from their teachers and/or peers in the *Intensive Reading* class. These questions were designed to stimulate the participants to recall their memories of activities or tasks in the stages of “motivating” and “enabling” in the POA-based *Intensive English Reading* course. In addition, this section also inquired the feedback and evaluation the interviewees received from their teachers and peers in the POA-based *Intensive Reading* course. This question was aimed to understand the influence of the stage of assessing.

### Data Collection

The participants were asked to complete the L2MSS questionnaires at the beginning and the end of the experiment, which lasted one semester. The survey was administered during the break of the class. Before the participants responded to the questionnaire, they were briefed on the purposes of this study and methods to answer. They were ensured that their responses would pose no threat to their final scores on the subject and would be kept confidentially and merely utilized for research purposes.

To obtain information on the attractors for change in the L2MSS of the participants, follow-up interviews were conducted on the 13 invited interviewees in the early, middle, and late stages of the semester. Before the interview, each interviewee was asked to fill in a copy of the questionnaire to record the level of their motivation at the time of the interview. The interviews were conducted in Chinese. Each interview lasted approximately 30 minutes and was recorded for later analysis.

### Data Analysis

The collected questionnaire data were processed with SPSS 26.0. In order to ascertain potential changes in L2MSS among the participants after the POA-based teaching experiment (Research Question 1), an independent *t*-test was performed between the EG and the CG.

To seek answers to the second research question with regard to the potential attractors that might have caused changes in the L2MSS among the participants after the POA-based teaching experiment, the data collected during the interviews were analyzed with the following steps. The independent *t*-test was first conducted on the answered L2MSS scales of the 13 interviewees to identify differences in the initial conditions of their motivation levels. Then, a qualitative analysis approach (Dörnyei, 2007) was followed to analyze the attractors responsible for changes in L2MSS among the participants with ATLAS.ti. When the authors completed transcribing the recorded interviews, the interviewees were invited to check their respective transcripts so as to guarantee the accuracy of the content they intended to express. Next, the authors carefully read and coded the transcripts, with particular attention paid to the attractors that might have led to changes in L2MSS among the interviewees. Any segment related to influence on L2MSS among the participants was coded. For example, the quote “*The teacher is guiding us to think deeply*” was coded as the attractor of “*Teacher Guidance*.” Through rigorous coding and repeated discussion among the authors, the coding of emerging themes was completed. The coded themes were rigorously discussed among the authors to ensure the external validity of the data analysis with those inconsistent coded results further analyzed. To guarantee the internal validity of data coding and credibility of the study, the coding result was shared with the interviewees for further revisions (Creswell, 2013).

## Results

### Changes in L2MSS Among the Participants

The independent *t*-test (Table 1) was conducted in order to identify possible changes in the overall and dimensional categories of L2MSS among the participants. It can be seen that there is no significant difference in the level of their L2MSS (*p* = 0.624 > 0.05), Ideal L2 Self (*p* = 0.753 > 0.05), and L2 Learning Experience (*p* = 0.202 > 0.05) before the experiment between the EG and the CG. However, they displayed a significant variation in Ought-to L2 Self (*p* = 0.04 < 0.05) before the experiment. It seems that the two groups, except for being significantly different in their Ought-to L2 Self, were equally motivated in their English language learning when the experiment was commenced.

**TABLE 1:** Second language motivational self system (L2MSS) between the two groups before and after the experiment.

		**L2MSS**		**Ideal L2 Self**		**Ought-to L2 Self**		**L2 Learning Experience**	
		**Before**	**After**	**Before**	**After**	**Before**	**After**	**Before**	**After**
Mean	Experimental group	3.02	3.69	3.48	4.44	2.07	2.22	3.6	4.57
	Control group	3.08	3.16	3.44	3.47	2.43	2.48	3.41	3.60
*t*		–0.493	5.456	0.317	7.017	–2.116	–1.405	1.292	7.451
*P* [sig. (two-tailed)]		0.624	0.000	0.753	0.000	0.040	0.167	0.202	0.000

With regard to their L2MSS after the experiment, the two groups showed sharp differences in the overall level of L2MSS (*p* = 0 < 0.05) and in two of the three dimensions, namely, Ideal L2 Self (*p* = 0 < 0.05) and L2 Learning Experience (*p* = 0 < 0.05). It is interesting to note that the two groups displayed no significant difference in Ought-to L2 Self (*p* = 0.167 > 0.05) after the experiment. The results indicate that after the POA teaching experiment the EG became significantly more strongly motivated than the CG who did not receive teaching with the POA model. In particular, the former was significantly more motivated by their Ideal L2 Self and L2 Learning Experience than the latter.

The L2MSS of the two groups before and after the experiment was compared for a further understanding of the influence of the POA model on the participants (Table 2). Significant differences can be observed in the overall level of L2MSS (*p* = 0.000 < 0.05), Ideal L2 Self (*p* = 0 < 0.05), L2 Learning Experience (*p* = 0 < 0.05) before and after the experiment among the EG, while they showed no significant difference in their Ought-to L2 Self (*p* = 0.489 > 0.05). These results revealed that the EG experienced a significant increase in their L2MSS after studying the POA-based *Intensive English Reading* course for a semester, although their Ought-to L2 Self did not significantly change. In contrast, the CG reported no significant differences in the overall level of their L2MSS (*p* = 0.421 > 0.05), Ideal L2 Self (*p* = 0.778 > 0.05), Ought-to L2 Self (*p* = 0.751 > 0.05), and L2 Learning Experience (*p* = 0.2 > 0.05). It implies that the L2MSS of the learners did not change obviously in the *Intensive English Reading* course with the traditional teaching model.

**TABLE 2:** L2MSS of the two groups before and after the experiment.

**Items**		**L2MSS**		**Ideal L2 Self**		**Ought-to L2 Self**		**L2 Learning Experience**	
		**CG**	**EG**	**CG**	**EG**	**CG**	**EG**	**CG**	**EG**
Mean	Pretest	3.08	3.02	3.44	3.48	2.43	2.07	3.41	3.6
	Posttest	3.16	3.69	3.47	4.44	2.48	2.22	3.60	4.57
*t*		–0.812	–5.801	–0.285	–6.486	–0.319	–0.697	–1.297	–7.773
*P* [sig. (two-tailed)]		0.421	0.000	0.778	0.000	0.751	0.489	0.200	0.000

### Attractors Responsible for Changes in L2MSS

#### Initial Conditions

System shows sensitive dependence on initial conditions (Larsen-Freeman, 2015), which means that a small change in initial conditions could exert profound influences on the future behaviors of an individual. To identify whether the two groups shared the same initial conditions, the interviewees were asked to fill in the L2MSS scale before the first round of interviews. The results of the independent *t*-test (Table 3) showed no significant differences in the overall level of L2MSS (*p* = 0.788 > 0.05), Ideal L2 Self (*p* = 0.703 > 0.05), Ought-to L2 Self (*p* = 0.527 > 0.05), and L2 Learning Experience (*p* = 0.827 > 0.05). That is, there is no significant difference in the initial conditions of the L2MSS between the two groups.

**TABLE 3:** L2MSS of the two groups before the first interview.

**Items**		**L2MSS**	**Ideal L2 Self**	**Ought-to L2 Self**	**L2 Learning Experience**
Mean	Experimental group (*N* = 8)	3.47	3.98	2.55	3.93
	Control group (*N* = 5)	3.38	4.10	2.22	3.84
*t*		0.276	–0.391	0.654	0.223
*P* [sig. (two-tailed)]		0.788	0.703	0.527	0.827

#### Attractors Responsible for Changes in L2MSS Among the Participants

The results of the interview data analyzed by following the qualitative content analysis approach (Dörnyei, 2007) showed a host of attractors that underlie the changes in the L2MSS of the participants. These attractors are found to be mainly associated with Ideal L2 Self and L2 Learning Experience. The attractors for Ideal L2 Self include *Output Tasks* in the motivating stage and *Peer Performance* in the assessing stage of the POA teaching model. The attractors for L2 Learning Experience entail *Output Tasks* in the motivating and assessing stages, *Teacher Guidance* in the motivating and enabling stages, *Group Discussion* in the enabling stage, and *Peer Assessment* in the assessing stage.

##### Attractors Affecting Ideal L2 Self

Ideal L2 Self represents the ideal feature that an individual seeks to possess and is related to positive results and emotions in language learning (Dörnyei, 2009). The quantitative results revealed a significant increase in Ideal L2 Self among the participants in the EG after the POA teaching experiment. The increase in their L2MSS might be caused by the growing depth and width of attractor basins throughout the POA teaching process, as reflected in the interviews.

The POA approach holds that the production objectives should be motivating, and teachable (Wen, 2017). The interviews revealed that *Output Tasks* were a critical attractor contributing to the increased Ideal L2 Self of the learners. As student C expressed as follows, “*Each unit we would have a task related to the unit, which was usually presented at the very beginning. However, since we had learned the important part of the text, we didn’t know what to do. I think I need to learn more if I want to do the task well*” (student C, interview I). In the beginning of the POA-based *Intensive English Reading* course, the teacher assigned the output tasks first. These tasks were usually slightly beyond the current level of the learners, thus meeting the requirements of appropriate difficulty in the motivating stage of the POA model (Duan, 2020). This challenge to complete the task, thus, made the students realize the gap between their actual ability and the ability needed to complete the task. It seems that the output task created a sense of “hunger” and stimulated the desire of the students to learn. The process of narrowing their gap on knowledge was the process of narrowing the gap between their actual self and ideal self.

Another attractor leading to the widening of the attractor basin of Ideal L2 Self pertains to *Peer Performance* in the assessing stage. The POA model advocates an immediate and collaborative assessment (Wen, 2018). During the assessing stage, the learners are supposed to deliver their results of the output tasks and receive immediate feedback from both peers and teachers. The immediate assessment, meanwhile, provides opportunities for those learners with lower levels of English proficiency to realize the gap between themselves and their higher-level peers. This is illustrated as follows: “*there was an invisible pressure when I saw that all the students in my class were quite good at speaking. I hope I could have excellent oral English as them*” (student B, interview II). Students with lower English proficiency would thus be stimulated to pursue an ideal image of equal fluency as those more proficient peers did.

##### Attractors Affecting L2 Learning Experience

L2 Learning Experience refers to the situational motivation that closely pertains to specific learning situations, such as curriculum setting, classroom activities, teacher behavior, and prior learning experience (Dörnyei, 2009; Chang, 2018). The quantitative findings suggested a strengthened intensity of L2 Learning Experience in the EG after attending the POA-based *Intensive English Reading* course for one semester. This might be explained by the attractors throughout the POA teaching process, as revealed in the interviews.

The *Output Tasks* in the POA-based class seemed to be a major driving force in enhancing the L2 Experience of the learners, as implied in the interviews. The wide variety of output tasks in the motivating stage effectively generated the interest of the learners in learning English as illustrated in the following excerpt: “*The debate was so interesting that everyone in our class was eager to take part in*” (student F, interview III). There were various output tasks, such as debate, performance, reading salon, poster presentation, mind maps, drama performance, and others assigned during the motivating stage in POA-based class. This rich variety of output tasks seemed to arouse the interest of the students and stimulated their desire to engage themselves into the tasks.

Moreover, the *Output Tasks* in the assessing stage seem to create opportunities for the learners to use their prior knowledge, as commented by one of the interviewees as follows: “*I think that was good. When reporting our task results to the class, I could apply the knowledge covered in the text of the unit, from which I could obtain a sense of harvest*” (student C, interview I). As acknowledged by the interviewee, the learners, while completing the output tasks, could get opportunities to practice what they had learned in the unit. This could make it more enjoyable and effective for them to learn English.

In addition, the *Output Tasks* in the assessing stage helped the learners envisage a future self. The output tasks in the POA model are designed to be closely related to communicative scenarios that the learners would encounter in their further study and life (Wen, 2018). As commented by student E, when she worked together with her group members to write a mini-novel on the topic “*The Love Story between Me and My partner*,” her group became much excited because “*The topic of this task was close to our lives, which made us have a lot of words to write. Our group discussed for quite a long time, and in the end we wrote a very long novel*” (student E, interview II). To complete these tasks might help the learners see the connection of the tasks with their personal needs, from which they can get a sense of their own future possibility of these scenarios.

In contrast, there were few real output tasks in the conventional class. An interviewee from the CG said that “*Usually, we would have a classmate assigned either to introduce the background of the text or anything that he/she was interested in. Apart from that, there were barely any other class tasks. So, I was not impressed with the presentation in class*” (student I, interview III). The learners from the untreated class were, thus, less strongly motivated by their L2 Experience.

Teachers play a crucial part throughout the POA teaching process (Tang, 2020). In the motivating stage, teachers designed the communicative tasks centering on teaching objectives (Wen, 2017). When the learners felt the cognitive challenge from the assigned tasks in the motivating stage, teachers would guide the learners to cultivate autonomy awareness and to think critically about the tasks. This is illustrated in the following excerpt that “*It may be a little bit difficult for me to get accustomed to this rhythm at the beginning of this class. Then, my teacher guided us to think how to solve these tasks by ourselves. After one class I felt that I have gained a lot, not only the knowledge learned, but also the critical thinking capacity*” (student G, interview III). The worry and anxiety of the learners seemed to be alleviated by the guidance of the teachers, from which they acquired an enjoyable learning experience.

Their joyful L2 Experience also stemmed from the scaffolding from the teachers in the enabling stage. As illustrated in the following excerpt, “*Once we were supposed to make a poster presentation on which we should include the key language points learned in the unit. It was difficult for me in the very beginning. Then, I turned to my teacher for help. My teacher explained the requirements of the task and clarified my confusion. It became much clearer to me and I won prize from him*” (student A, interview II). The teacher provided support of knowledge and skills required to complete the tasks of the learners (Wen, 2017). The scaffolding facilitated the learner to complete the task better and increased her sense of enjoyment in this learning experience.

On the contrary, teachers in the traditional class seemed to fail in bringing positive L2 Experience to the learners. As student K expressed, “*In class, the teacher usually processed the text first, and then explained the author’s writing intention. The vocabulary and grammar points were also explained one by one. I didn’t think there was any challenge*” (student K, interview II). It is revealed that the teacher dominated the traditional class, which exerted a scant influence on the interest of the students in learning English.

*Group Discussion* is another attractor of L2 Learning Experience in the enabling stage. Collaborative learning in groups is one of the features of the POA model in the enabling stage (Wen, 2018). As mentioned by student B that “*We could extend a lot each time when we discussed in groups, and I think that was pretty good. I could also learn about others’ ideas and find out that a problem could be thought of from so many angles, some of which I really wouldn’t realize*” (student B, interview III), students can exchange their ideas and learn from each other through group discussions. When engaged into group discussions, the learners could practice their spoken English and intensify their sense of achievement in using English. In contrast, students in the CG reported that they had few group discussions in the class.

Finally, *Peer Assessment* is also an important attractor of L2 Learning Experience in the assessing stage. POA believes that the integration of assessment into the learning process can help learners understand their learning better and, thus, lead to better results (Wen, 2018). “*Once in a group peer assessment activity, a classmate pointed out that my essay had too many long sentences, which she thought too tedious. I was really appreciative of that, because I didn’t actually realize when I was writing*” (student G, interview II). Through the assessment process, students can realize the strengths and weaknesses of their output works. From this process, they could gain a sense of accomplishment, which enhances their L2 Learning Experience. In contrast, interviewees from the CG claimed that there was basically no assessment session in their class.

## Discussion

This study first examined the changes in L2MSS among the participants. It has been found that the EG experienced a significant increase in the overall level of L2MSS and in Ideal L2 Self and L2 Learning Experience. They also displayed significantly stronger L2MSS, Ideal L2 Self, and L2 Learning Experience than the CG. In contrast, the untreated CG showed no significant changes in the overall and dimensions of the L2MSS construct. The findings suggested that the POA-based model has a positive impact on the L2MSS of the learners, especially on their Ideal L2 Self and L2 Learning Experience. Ought-to L2 Self, however, was hardly affected by the POA model. These findings lend support to previous studies that POA model can be effective in promoting the L2MSS of EFL learner, their Ideal L2 Self and L2 Learning Experience in particular (Wang and Dai, 2015; Duan, 2020).

These results indicate that the attractor basin of Ideal L2 Self and L2 Learning Experience significantly deepens and widens, while the attractor basin of Ought-to L2 Self basically remains unchanged among the participants after the treatment of the POA teaching model. According to the DST, attractors drive the change in a system. The scale of attractors determines the range of L2 motivational fluctuation (Chang, 2019). The interview data suggested that there were more attractors of Ideal L2 Self than attractors of L2 Learning Experience in the POA-based *Intensive English Reading* course. Therefore, the attractor basin of L2 Learning Experience became deeper than the attractor basin of Ideal L2 Self.

Analysis of the qualitative data revealed that the attractors leading to change in L2MSS among the participants mainly lie in two attractor basins of Ideal L2 Self and L2 Learning Experience. The widening of the attractor basin of Ideal L2 Self might be caused by the *Output Tasks* in the motivating stage and *Peer Performance* in the assessing stage of the POA model. *Output Tasks* assigned in the motivating stage set the goals for the learners. These goals are often beyond the current level of the learners, thus posing a certain cognitive challenge to them. This challenge would make the learners come to realize the gap between what they know and what they have not known. The goals to complete tasks functioned as a direction, as expressed by some interviewees, of the efforts of learners and investment into pursuing their future self. These goals are the vision of Ideal L2 Self, which shall decide subsequent learning behaviors (Dörnyei, 2009; Chang, 2018). When the Ideal L2 Self of the learners is to command the target language, a powerful driving force prompted by the mastery goals as a positive L2 self would thus be generated for the learners to strive to shorten the gap between their actual self and ideal self (Chang, 2018; Chen et al., 2020).

*Peer Performance* in the assessing stage of the POA model is another attractor that contributes to the deepening of the attractor basis of Ideal L2 Self. Positive Psychology posits that positive traits such as hope and resilience, could expand thinking capacity (Seligman and Csikszentmihalyi, 2000; MacIntyre et al., 2016). When learners felt pressure from the excellent performance of peers, their resilience seemed to help them to turn this peer pressure into facilitative anxiety, as reflected by student B. Thus, a positive Ideal L2 Self is constructed (Chang, 2019). This positive Ideal L2 Self works as a driving force directing the efforts and behaviors of learners to generate, maintain, and even strengthen their L2 motivation (Xu and Fang, 2016; Chang, 2019).

Regarding the widening of the attractor basin of L2 Learning Experience, this research found that *Output Tasks*, *Teacher Guidance*, *Group Discussion*, and *Peer Assessment* throughout the POA teaching process nurture a positive learning experience among the interviewees. According to Positive Psychology, positive experience, such as enjoyment, happiness, interest, and satisfaction, contributes to the expansion of attentiveness and thinking mode (Seligman and Csikszentmihalyi, 2000). This positive experience leads to the broadening of horizon and strengthening of resilience among the learners (Fredrickson, 2001). A happy and interesting learning experience would turn to an attractor of an L2 motivational system (Chang, 2019). The positive experience then deepens the attractor basin of L2 Learning Experience.

The *Output Tasks* were a major attractor in shaping positive experience among the interviewees. The POA model is featured with a rich variety of output tasks. This wide variety arouses the interest of the learners and creates opportunities for them to apply their prior knowledge into practice (Wen and Sun, 2020). Putting the prior knowledge learned previously from the completion of the output tasks could prompt the learners to experience enjoyment and give them a sense of success. Finding learning a language interesting and enjoyable echoes Chen et al.’s (2020) interest-in-L2 self. The topics and contents of the output tasks are designed to be related to the practical needs of the learners. This real-life communication orientation could help the learners see the connection between what they are learning and what they could get for their future image. This sensed connection could prompt the learners to generate a passion for L2 learning and envisage a future self (Chen et al., 2020). This envisaged self would mold their positive attitude toward English learning and increase their motivation. By contrast, a lack of these various output tasks might fail to impress the learners with less enjoyable learning experience.

Teachers are critical agents throughout the POA teaching process (Sun, 2020). They design communicative scenarios directed by the teaching objectives (Wen, 2018). As expressed by some of the interviewees, the confusion and anxiety of the learners in completing the output tasks in the motivating stage seemed to be alleviated by the guidance and support of the teacher, from which they acquired an enjoyable learning experience (De Ruiter et al., 2019). Their joyful L2 Experience was also attributable to the scaffolding from the teachers in the enabling stage. Support of knowledge and skills required to complete the tasks to the learner would be provided by the teacher (Yang and Yu, 2017). The scaffolding benefited the learners to succeed in completing the output tasks, from which they could harvest a sense of enjoyment in this learning experience. On the contrary, the teachers in the traditional class failed in bringing a positive L2 Experience to the learners.

*Group Discussion* is found to be another attractor of L2 Learning Experience in the enabling stage. The POA model encourages collaborative learning through output tasks and projects in the enabling stage (Wen, 2018). Students can exchange their ideas and learn from each other through group discussions. When collaborating in group activities, learners could share ideas with their peers and form a harmonious relationship inside and outside class, which increases their sense of achievement and enjoyment of language learning (Elahi Shirvan et al., 2020). Interest in these output tasks drives the learners to invest their effort and attention to L2 learning, and strengthens their willingness to participate in a group discussion. Increased effort and enjoyment, thus, enhance their motivation to learn English (Teimouri et al., 2020). In contrast, students in the CG were exposed to limited group discussions in the class.

The L2 Experience of the learners is also strengthened by the attractor of *Peer Assessment* in the assessing stage. Collaborative assessment between teacher and peers is integrated into class in the POA model (Qiu, 2020). The interaction in peer assessment offers an opportunity for the learners to reflect on their advantages and disadvantages in their language learning. From this process, they gradually develop a rational attitude toward themselves and obtain a sense of accomplishment. This felt sense of fun, pride, and progress could help them experience joy and form a positive language learning experience (Elahi Shirvan et al., 2020; Wang et al., 2021). In contrast, interviewees from the CG claimed that there was basically no assessment session in the class.

## Conclusion

This study examined the effect of POA-based model on L2MSS of Chinese university English majors from a DST perspective. It was found that the POA model is effective in strengthening L2MSS among the participants, Ideal L2 Self and L2 Learning Experience in particular. This result indicates the dynamic nature of L2MSS and its context-responsive feature (Dörnyei et al., 2015). Besides, the strengthened Ideal L2 Self of the participants is caused by an attractor basin that is deepened by a number of attractors, such as *Output Tasks* and *Peer Performance*. In addition, the increased L2 Learning Experience of the learners is associated with an attractor basin consolidated by a host of attractors that contain *Output Tasks*, *Teacher Guidance*, *Group Discussion*, and *Peer Assessment*. The positive experience promotes a belief among the learners in the attainability of language acquisition (Mercer and Ryan, 2010). It also nurtures a belief that persistence and effort could contribute to L2 learning competence, enhance confidence, and boost motivation (Brown and Seibert Hanson, 2019). These findings lend support to the opinion that a dynamic system is associated with subjective and social dimensions (Dewaele and MacIntyre, 2016). This study suggests that L2MSS development is closely related to complex, dynamic, self-organizing, and adaptive systems (Larsen-Freeman and Cameron, 2008). It enriches the present literature on L2 motivation in a POA-based context from a DST perspective and indicates the possibility of examining L2 motivation from a Positive Psychology lens.

This research has certain pedagogical implications. Teachers should pay attention to the selection of topics as well as the variety of output tasks in class. A rich variety of output tasks helps to attract the attention of students and enhance their engagement in English learning. Authentic tasks relevant to the study and life of students would be effective in generating interest of students and boost the confidence in completing the tasks. Then, it is imperative for teachers to play a scaffolding role and act as guide to promote effective learning. Last but not the least, assessment needs to draw due attention as a pedagogical method. For teachers, assessment allows them to keep track of the knowledge application of students, based on which they can adjust their teaching in time. For students, they can check whether they have used language points correctly through feedback from the teacher and their classmates. Besides, training should be provided to students in collaboration with peers in group activities and peer assessments, so that they can make use of interaction with more competent peers.

Several limitations should also be recognized. First, there are many motivational factors that influence language learning; but this study only focused on the factor of teaching mode. Future studies can expand the scope by taking more variables into account. Second, this study found that Ought-to L2 Self seemed not to be influenced by the POA teaching experiment. More studies are also called for to examine this aspect. Third, this study indicated the positive experience of learning in the POA model. Future research is suggested to explore this positive experience from a Positive Psychology perspective.

## Data Availability Statement

The original contributions presented in the study are included in the article/supplementary material, further inquiries can be directed to the corresponding author.

## Ethics Statement

The studies involving human participants were reviewed and approved by the School of Foreign Languages, Hubei University of Technology, China. The participants provided their written informed consent to participate in this study.

## Author Contributions

CL designed the study and revised and finalized the manuscript. CZ collected and processed the data and prepared the first draft. WZ processed and revised the draft. All authors contributed to the article and approved the submitted version.

## Conflict of Interest

The authors declare that the research was conducted in the absence of any commercial or financial relationships that could be construed as a potential conflict of interest.

## Publisher’s Note

All claims expressed in this article are solely those of the authors and do not necessarily represent those of their affiliated organizations, or those of the publisher, the editors and the reviewers. Any product that may be evaluated in this article, or claim that may be made by its manufacturer, is not guaranteed or endorsed by the publisher.
